# Psychosocial assessment tools for use before transplantation are predictive of post-operative psychosocial and health behavior outcomes: a narrative review of the literature

**DOI:** 10.3389/frtra.2023.1250184

**Published:** 2023-09-07

**Authors:** Sorin Thode, Keith Perry, Samuel Cyr, Anique Ducharme, David Puissant, Judith Brouillette

**Affiliations:** ^1^ Research Center, Montreal Heart Institute, Montreal, QC, Canada; ^2^ Faculty of Medicine, Université de Montréal, Montreal, QC, Canada; ^3^ Faculty of Pharmacy, Université de Montréal, Montreal, QC, Canada

**Keywords:** transplantation, psychosocial assessment tools, post-transplant outcomes, narrative review, PRISMA (preferred reporting items for systematic reviews and meta-analyses)

## Abstract

**Introduction:**

In end-stage diseases, transplantation may be necessary. The limited number of donors led to the development of several pre-transplant psychosocial assessment tools. We summarized the predictive value of these tools before solid-organ transplantation.

**Methods:**

The PRISMA search strategy and the MEDLINE database were used to review the literature. From 1,050 records, we found thirteen studies using four different scales (Millon Behavioral Health Inventory [MBHI], Psychosocial Assessment of Transplant Candidates [PACT], Stanford Integrated Psychosocial Assessment for Transplantation [SIPAT], and Transplant Evaluation Rating Scale [TERS]).

**Results:**

TERS and MBHI were associated with the highest number of positive studies concerning pre-transplant scores and primary outcomes. Psychosocial scales predict in a systematic way psychosocial and health behavioural outcomes, but generated mixed results for mortality and rejection.

**Discussion:**

This narrative review underlines the need for multidisciplinary evaluation and well-conducted clinical trials to assist transplant teams in utilizing psychosocial evaluation effectively during evaluation of candidates.

## 1. Introduction

Solid-organ transplantation offers life-saving treatment to patients suffering from end-stage organ dysfunction. However, the growing number of patients on the waiting list largely outweighs the number of donors’ organs available, a persistent concern in transplantation medicine. In the United States, although more than 40,000 transplants were achieved in 2022, the waiting list still comprised more than 100,000 candidates in need of an organ (1). In Canada, where our team is based, in 2021 alone, more than 4,000 patients were on a waiting list to receive a transplant, and 38% of them ultimately died while waiting (2, 3). The COVID pandemic, beginning in March 2020, has also had a negative impact on transplant success. Indeed, in Canada the total number of solid organ transplants has dropped by 14% from 2019 to 2020 (2, 3).

Due to the scarcity of resources and complexity of the treatment regimen surrounding transplant, potential candidates usually undergo a thorough preoperative screening. This allows for a precise understanding of the patient's global health status to determine whether they are suitable candidates for transplantation. Amongst these variables, psychosocial factors are now widely recognized health determinants (4), which are associated with transplantation outcomes (5, 6). Even though the Centers for Medicare & Medicaid Services (CMS) demands that a psychosocial examination is done before transplant (7), there is no solid research data supporting this practice. Different guidelines also agree on the necessity of screening patients for psychosocial risk factors before transplantation and this is usually done by a psychiatrist, psychologist and/or a social worker working as a part of the transplant team (8, 9). However, while the medical evaluation of transplant candidates is well standardized across hospitals, the evaluation method for psychosocial risk factors differs greatly. The instruments used for this psychosocial evaluation differ in their focus, with some assessing overall psychosocial functioning while others only measuring specific aspects (e.g., alcohol/drug use, depression, memory), providing a narrower focus. Nghiem et al.'s review of psychosocial assessment instruments for liver and kidney transplant candidates further confirms the scarcity of research on the psychometric properties of these tools and the need to investigate their effectiveness in predicting post-transplantation outcomes (6).

We thus conducted this narrative literature review to summarize the findings on psychosocial assessment tools used before either solid-organ (heart, lung, kidney, and liver) transplantation and their predictions of postoperative outcomes. Although transplant teams might use specific diagnostic tools for different conditions such as depression, substance use or other diseases that may impact transplant outcomes, these were not assessed in this study.

## 2. Methods

The PRISMA search strategy was used as a model to conduct this narrative review (10).

### 2.1. Eligibility criteria

The study sample included adults (age ≥ 18), solid-organ (heart, lungs, liver, or kidney) recipients and had to assess psychosocial functioning before transplantation. Psychosocial assessment tools had to be clearly and accurately specified. Table 1 represents an overview of the psychosocial assessment tools used to evaluate potential transplantation candidates. Scales used were Millon Behavioral Health Inventory (MBHI) (11, 12), Millon behavioral medicine diagnostic Instrument (MBMD) (12, 13), Psychosocial Assessment of Transplant Candidates (PACT) (14, 15), Stanford Integrated Psychosocial Assessment for Transplantation (SIPAT) (16), Structured Interview for Renal Transplantation (SIRT) (17), and Transplant Evaluation Rating Scale (TERS) (18). We excluded studies or publications that were literature or systematic reviews.

**Table 1:** Psychosocial pre-transplant assessment tools.

Questionnaire	#Items	Domains	Method of administration	Scoring
MBHI Millon Behavioral Health Inventory	150	1. Personnality style 2. Psychogenic attitude 3. Psychosomatic correlate 4. Prognostic index	Self-report	Coping style scales: Conversion of raw scores into base rate scores using prevalence data. Score above 74 indicated the presence of the particular characteristic that each scale represents. Psychogenic attitude scales: Transformed prevalence data into classical T scores.
MBMD Millon behavioral medicine diagnostic Instrument	165	1. Response Patterns 2. Negative Health Habits 3. Psychiatric Indications 4. Coping Styles 5. Stress Moderators 6. Treatment Prognostics 7. Management Guides	Self-report	Raw scores are converted to initial prevalence scores. Adjustment to the initial prevalence scores are then calculated to determine the final prevalence scores to plot on the patient's profile sheet.
PACT Psychosocial Assessment of Transplant Candidates	8	1. Social support 2. Psychological health 3. Lifestyle factors 4. Comprehension of transplant and follow-up	Clinician interview	Eight items rated on a five-point scale plus the evaluator's integration of all items into one final score: 0 (poor candidate), 1 (borderline candidate), 2 (acceptable candidate), 3 (good candidate), and 4 (excellent candidate). The final rating involving clinical judgement can overrule the total score.
TERS Transplant Evaluation Rating Scale	10	1. Prior psychiatric history with axis 1 disorders 2. Prior psychiatric history with axis 2 disorders 3. Substance use/abuse 4. Compliance 5. Health behaviors 6. Quality of family and social supports 7. Prior history of coping 8. Coping with disease and treatment 9. Quality of affect 10. Mental status (past and present)	Clinician interview (Typically 2 separate examiners)	Ten items rated on a three-point scale with a relative weight attributed to each item for calculation of a final weighted summary score. Higher score indicates lower level of psychosocial functioning. Individual sub scales results can also be reported. Higher score indicates lower level of psychosocial functioning.
SIPAT Stanford Integrated Psychosocial Assessment for Transplantation	18	1. Patient's Readiness Level and Illness Management 2. Social Support System Level of Readiness 3. Psychological Stability and Psychopathology 4. Lifestyle and Effect of Substance Use	Clinician interview	Candidates divided into 5 groups based on total score: Excellent (0–6), Good (7–20), Minimally Acceptable (21–39), Poor (40–69), and High Risk (70). Certain items more heavily weighted based on evidence that they are more predictive of clinical outcomes.
SIRT Structured Interview for Renal Transplantation	93	1. Background/Demographics 2. Understanding of Illness 3. Education/Socioeconomic Status 4. Brief Family History 5. Coping/Personality Style 6. Psychiatric History 7. Mental Status Exam	Clinician interview	The SIRT is not used as a stand-alone assessment tool. Clinicians review the patient's clinical chart, administer the SIRT and other psychometric testing (PACT) to write a final report about the patient's psychiatric appropriateness for transplantation.

### 2.2. Search strategy and study selection

**Figure 1:**
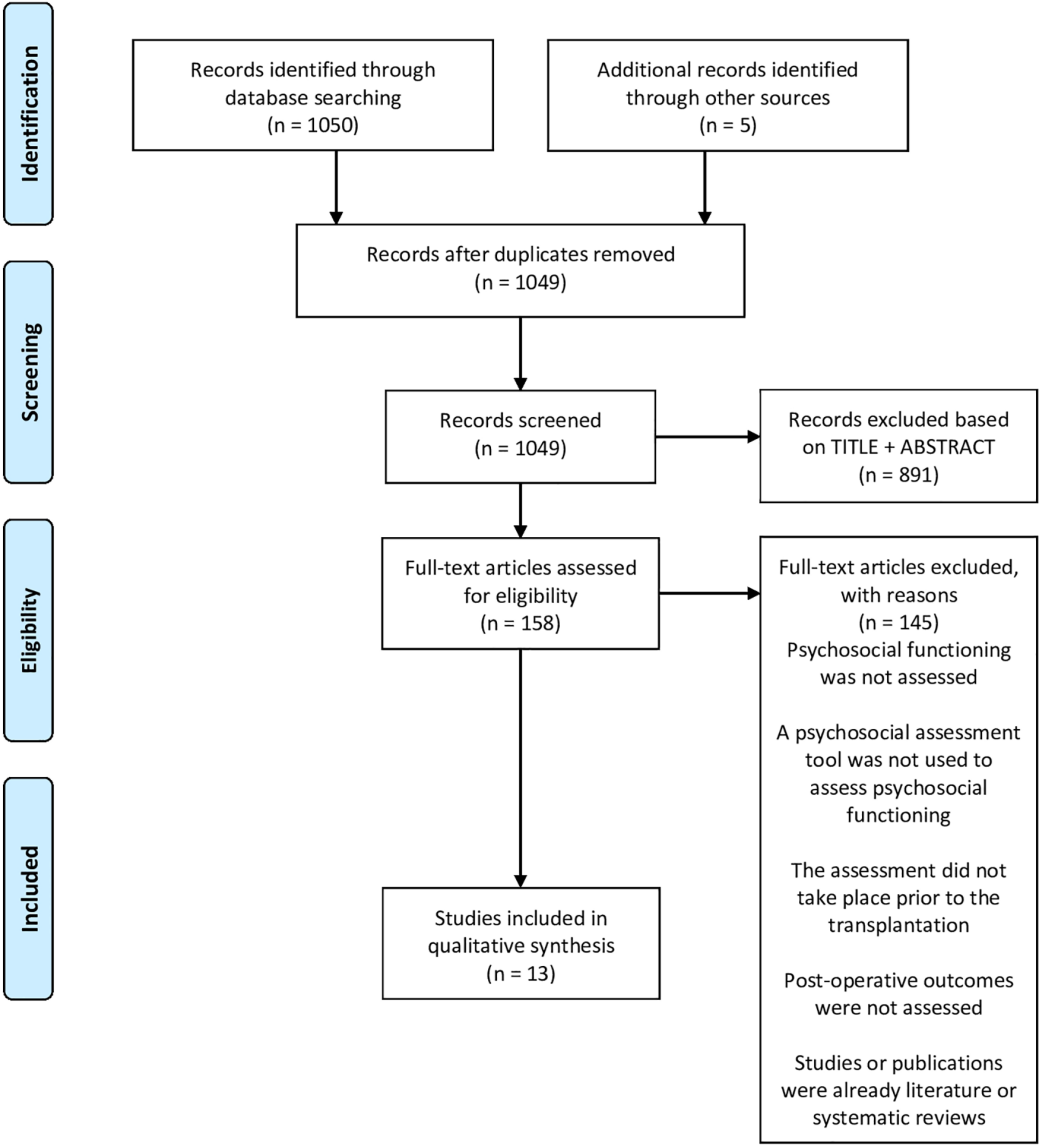
PRISMA flow diagram of literature review. visit www.prisma-statement.org

We identified publications through a search of MEDLINE and restricted results to publications in English or French. We present the exact search strategies and details as Supplementary Material. We performed the first search on July 28, 2020, with updates performed on June 09, 2021, and February 02, 2022. Figure 1 represents the PRISMA flow diagram illustrating the study selection process (10). Among the articles, we examined literature reviews and meta-analyses for valuable references. Titles and abstracts retrieved were then screened for eligibility by authors S.T., K.P. and S.C. using the established criteria. Publications with an unclear inclusion status after screening were discussed by authors S.T., K.P. and S.C. and either included or excluded.

### 2.3. Quality assessment

Authors S.T., K.P. and S.C. independently assessed the risk of bias for each study using the National Institute of Health's Quality Assessment Tool for Observational Cohort and Cross-Sectional Studies (National Institutes of Health [NIH] & National Heart, Lung, and Blood Institute [NHLBI] (19)). This tool consists of 14 criteria answered using Yes/No/Other (cannot determine, not applicable, not reported) that lead to the study's overall quality rating (good/fair/poor). There is no NHLBHI consensus on classifying articles into different categories. The authors subsequently discussed each article for classification. All thirteen articles were deemed to be of good quality and thus included.

## 3. Results

### 3.1. Study selection and characteristics of included studies

We found a total of 1,050 articles through the searches of the MEDLINE database. We retrieved five articles with literature reviews references, increasing the number to 1,055 potential articles, of which 1,049 remained after removing duplicates. Eight hundred ninety-one studies (84.9%) were excluded based on the title and abstract, and 145 (13.8%) were further excluded after a full-text examination using the established eligibility criteria. Our narrative literature review is thus including thirteen articles (1.2%). The selected items were either retrospective or prospective cohort studies.

### 3.2. Psychosocial assessment tools and post-transplantation outcomes

Table 2 summarizes the current state of knowledge on the ability of psychosocial tools to predict post-transplantation outcomes based on the thirteen accepted studies. We identified studies for only four of the six questionnaires presented in Table 1. It implies that two questionnaires, the MBMD and the SIRT, had not been studied in the current literature research in terms of outcome prediction.

**Table 2:** Information about included studies by pre-transplant psychosocial assessment tool.

Study Design, *N*	Variable type	*Organ*	Demographics	F-up period (Median) *Mean ± SD*	Primary outcome(s); main results	
*MBHI*						
Chacko et al. (20) Prospective f-up, *N* = 94	**Categ.**: Composite score of vulnerability using subscales	*Heart*	54 ± 12 y. 86% male	**56 months**	↓Survival time; *p* = 0.0004	
Harper et al. (21) Prospective cohort, *N* = 90	**Categ.**: High- vs. low-risk based on the median split of ranked MBHI score	*Heart*	53 ± 11 y. 86% male	**56 months**	↓Post-transplant survival time; *p* = 0.007	
Coffman et al. (22) Prospective cohort, *N* = 103	**Categ.**: Cut-off of 70, *Life Threat Reactivity*	*Heart*	–	**5 y.**	↔Mortality; *p* > 0.01	
Brandwin et al. (23) Prospective cohort, *N* = 103	**Categ.**: High- vs. low-distress	*Heart*	49 ± 10 y. 82% male	**1–5 y.**	↑Mortality; 5-year point: *p* < 0.005	
*PACT*						
Hitschfeld et al. (15) Retrospective cohort, *N* = 110	**Categ.**: <2 vs. ≥2	*Lung*	56 ± 11 y. 48% male	**12-y.** (3.6)	↑Mortality; aHR^a^ = 2.73 [95% CI: 1.07–7.01, *p* = 0.04]	
Schneekloth et al. (24) Retrospective cohort, *N* = 164	**Categ.**: <2 vs. ≥2	*Heart*	53 ± 11 y. 72% male	**12-y.** (7)	↔Survival	HR = 1.42; *p* = 0.6
	**Cont.**: 0–4					HR = 0.80; *p* = 0.3
Schneekloth et al. (25) Retrospective cohort, *N* = 538	**Categ.**: <2; 2–3; ≥3	*Liver*	54 ± 9 y. 70% male	**4–16 y.**	↔Survival overall	PACT (categorized; PACT ≥3 as reference); *p* = 0.4 PACT < 2; HR = 1.13 [95% CI: 0.78–1.65, *p* = 0.5] PACT 2–3; HR = 1.25 [95% CI: 0.89–1.77, *p* = 0.2]
	**Cont.**: 0–4					HR = 0.90 [95% CI: 0.77–1.05, *p* = 0.2]
*SIPAT*						
Maldonado et al. (5) Prospective cohort, *N* = 217	**Cont.:** Total SIPAT score	*Solid*	52 ± 13 y. 60% male	*341 ± 80 days*	↔Organ failure; HR = 0.98 [95% CI: 0.92–1.06, *p* = 0.7]^b^ ↔Mortality; HR = 0.99 [95% CI: 0.96–1.04, *p* = 0.8]^b^	
Deutsch-Link et al. (26) Retrospective cohort, *N* = 61	**Categ.:** <21; ≥21	*Liver*	–	(38 months IQR: 24–56)	↑Alcohol relapse; HR = 6.40 [95% CI: 1.36–30.18, *p* = 0.02]	
Deutsch-Link et al. (27) Retrospective cohort, *N* = 371	**Categ.:** <21; ≥21	*Liver*	59 y. [IQR 52; 64] 72% male	**2–5 y.**	↑Immunosuppression nonadherence; aOR = 2.92 [95% CI: 1.69–5.03, *p* < 0.001]	
	**Categ.:** *Patient's Readiness Level*				↑Biopsy-proven rejection; aOR = 2.66 [95% CI: 1.20–5.9, *p* = 0.02]	
Becker et al. (28) Retrospective cohort, *N* = 182	**Cont.**: SIPAT total score	*Liver*	56 ± 11 y. 67% male	*3 ± 1 y.*	↔Rejection of transplant; OR = 1.01 [95% CI: 0.97–1.06, *p* = 0.7]	
*TERS*						
Twillman et al. (18) Retrospective f-up, *N* = 35	**Cont.**: Total TERS score	*Liver*	48 ± 10 y. 25% male	**1–3 y.**	5 subscales of Visual Analogue Scale^b^ ↓Compliance r = −0.636; *p* < 0.001 ↑Substance use r = 0.643; *p* < 0.001 ↓Health behaviours (exercise, no smoking, diet, etc.) r = −0.671; *p* < 0.001 ↓Quality of life r = −0.415; *p* = 0.03 ↔success of orthotopic liver transplant r = −0.227; *p* = 0.2	
Baranyi et al. (29) Retrospective f-up, *N* = 123	**Cont.**: Total TERS score	*Solid*	53 ± 12 y. 70% male	*25 ± 12 months*	↑Level of overall mental distress; *p* = 0.03	

↑, Significant increase in variable/outcomes measure; ↓, Significant decrease in variables/outcome measure; ↔, No significant association; 95% CI, 95% confidence interval; Coef., Coefficient; f-up, follow-up; HR/aHR, hazard ratio/adjusted hazard ratio; IQR, interquartile range; MBHI, millon behavioral health inventory; MR, mean ratio; OR/aOR, odds ratio/adjusted odds ratio; PACT, psychosocial assessment of transplant candidates; SE, standard error; SIPAT, stanford integrated psychosocial assessment for transplantation; Solid organ includes heart, liver, lung/heart and lung; TERS, transplant evaluation rating scale; y., years.

^a^ Adjusted for sex, age, pulmonary vascular disease, and bilateral lung transplantation.

^b^ Despite the presence of multiple primary outcomes, the authors do not mention any control for type I error rate (alpha partition).

### 3.3. Million behavioural health inventory (MBHI)

Four of thirteen studies (20–23) used the MBHI for evaluation of heart transplant candidates. This makes it the most studied psychosocial scale, tied with the SIPAT. In all of them, the MBHI was treated as a categorical variable, with low or high-risk groups.

All studies assessed either post-transplant survival/mortality or survival time as a primary outcome, with three out of four reporting MBHI as a significant predictor (20, 21, 23). Indeed, Chacko et al. (20) showed that the specific factor-analyzed composite measure of vulnerability for the Millon scale significantly predicted the survival time (*χ*^2^ = 12.53, *df *= 1, *p *= 0.0004). In another study, Harper et al. (21) also showed the ability of the MBHI to predict longer survival time in participants in the low-risk group (*χ*^2^ = 7.24, *df *= 1, *p *= 0.007). Brandwin et al. (23) identified a significant association between mortality and the high-distress cluster over a 5-year period (status after 1 year: *χ*^2^ = 8.93, *df *= 1, *p* < 0.005/status after 5 years: *χ*^2^ = 8.16, *df *= 1, *p* < 0.005). Coffman & Brandwin (22) was the only team that did not find a significant association between MBHI and mortality between groups (*χ*^2^ = 2.35, *df *= 1, *p *> 0.01).

### 3.4. Psychosocial assessment of candidates for transplant (PACT)

Three out of thirteen studies used the PACT to assess psychosocial functioning in lung (15), heart (24), and liver (25) transplant candidates. The PACT was treated as only a categorical variable in one (13), and both a categorical and continuous variable in the two other studies (24, 25).

Three studies assessed survival/mortality as a primary outcome. A clear association between PACT score and higher mortality after lung transplantation was only found by Hitschfeld et al. (15), after adjustment with sex, age, pulmonary vascular disease, and bilateral lung transplantation (aHR = 2.73 [95% CI: 1.07–7.01, *p* = 0.04). In contrast, two studies from Schneekloth et al., (one in heart transplant and the other with liver transplant), reported no association between categorical and continuous PACT scores and survival (24, 25). However, in their liver transplant study (25), when studying a multivariable model with age at liver transplant, pre-transplant BMI, and marital status, women with a lower PACT score had significantly worse survival (HR = 0.64 [95% CI: 0.47–0.86, *p* = 0.003].

### 3.5. Stanford integrated psychosocial assessment for transplantation (SIPAT)

Four out of thirteen studies employed the SIPAT to assess psychosocial functioning in heart, lung, liver, and kidney (5), and liver (26–28) transplant. This makes it the most studied psychosocial scale, on par with the MBHI. The SIPAT was interpreted as either a continuous or a categorical variable, with a higher score representing a higher psychosocial risk.

Mortality was assessed as a primary outcome in a study by Maldonado et al. (5), but no association with pre-transplant SIPAT score was found [HR = 0.99 (95% CI: 0.96–1.04, *p* = 0.8)]. While three studies looked at graft failure or rejection, only Deutsch-Link et al. (27) found an association between the specific subdomain 1 (Patient's Readiness Level) and a higher risk of rejection after 3 months [aOR = 2.66 (95% CI: 1.20–5.91, *p* = 0.02)]. Maldonado et al. (5) [HR = 0.98 (95% CI: 0.92–1.06, *p* = 0.65] and Becker et al. [OR = 1.01 (95% CI: 0.97–1.06, *p* = 0.66)] did not find any significant association.

Deutsch-Link et al. (26) showed that a SIPAT score ≥ 21 (minimally acceptable to poor psychosocial risk) was significantly associated with post-transplant alcohol relapse [HR 6.40 (95% CI: 1.36–30.18, *p* = 0.02)] following liver transplantation and a SIPAT score ≥21 was significantly associated with lower adherence to immunosuppressive regimen [aOR 2.92 (95% CI: 1.69–5.03, *p* < 0.001)] in cardiac recipients (27).

### 3.6. Transplant evaluation rating scale (TERS)

The TERS was used to evaluate the psychosocial functioning of transplant candidates [liver (18) and heart, liver, or lung (29)] in two of the thirteen studies. The studies treated TERS score as a continuous variable.

Baranyi et al. showed that higher pre-transplant TERS score was associated with overall significant mental distress post-transplantation [Mann-Whitney-U = 1.255; *p* = 0.033] (29). In addition, Twillman et al. revealed that pre-transplant TERS score significantly correlated with levels of compliance, substance abuse, health behaviours, and quality of life when measured 1–3 years after transplant (18).

## 4. Discussion

We reviewed the literature on psychosocial questionnaires used to assess preoperatively solid-organ and their potential utility in predicting different post-transplantation outcomes. We herein outlined the different tools available and summarize the results of published studies on their predictive ability.

The first observation is the relative scarcity of available data on this topic despite the almost universal use of these surveys in clinical practice, a finding that has also been previously recognized by Nghiem et al. (6). Indeed, out of the six psychosocial questionnaires presented in Table 1, only four had available evidence on their pre-transplant use and association with postoperative outcomes. Consequently, two potentially relevant scales, the MBMD and the SIRT, could not even be included in this narrative review. Although these two older questionnaires, from the early 2000s, may be considered outdated compared to newer tools such as the SIPAT or the PACT, described in the late 2010s, it is still noteworthy that no investigator has ever focused on their predictive aspect. Also, of the remaining four questionnaires ultimately included in this literature review, we found an average of only three reports per questionnaire (ranging from two to four), highlighting the need for more research on this understudied field despite its wide clinical acceptance.

Overall, as summarized in Table 3, of the thirteen studies included, nineteen primary outcomes were verified, and twelve were significantly associated with the results of the pre-transplant evaluation, demonstrating that the predictive capacity of psychosocial scales is good but somewhat imperfect. However, it is worth noting various elements of these included articles that may have an impact on their conclusions. First, it should be noted that the various studies included did not have a standardized methodology, which also limits the conclusions that can be drawn from them. Also, the calculation of the sample size necessary to observe a significant difference (the power) was not presented in many of the different studies, which render difficult the determination of whether the absence of significance is due to a lack of power or to a real lack of predictive capacity of the scale. Furthermore, despite the recognition that choosing a threshold that maximizes sensitivity and selectivity is paramount for identifying patients at risk (30), evidence for the choice of the threshold is not always mentioned. An inadequate threshold could therefore weaken prediction of post-transplant outcomes.

**Table 3:** Summary of the different assessed primary outcomes with emphasis on statistical significance.

Medical outcomes			Psychosocial and health behaviours outcomes		
Mortality			Overall mental distress		
MBHI Brandwin et al. (23)	S *p* < 0.005	Heart	TERS Baranyi et al. (29)	S *p* = 0.03	Solid organ (heart, liver, lung/heart and lung)
MBHI Coffman et al. (22)	NS *p* > 0.01	Heart			
PACT Hitschfeld et al. (15)	S *p* = 0.04	Lung			
SIPAT Maldonado et al. (5)	NS *p* = 0.8	Solid organ (heart, lung, liver, kidney)			
Survival time/Survival/Days to death			Quality of life		
MBHI Chacko et al. (20)	S *p* = 0.0004	Heart	TERS Twillman et al. (18)	S *p* = 0.03	Liver
MBHI Harper et al. (21)	S *p* = 0.007	Heart			
PACT Schneekloth et al. (24)	NS Categorical: *p* = 0.6 Continuous: *p* = 0.3	Heart			
PACT Schneekloth et al. (25)	NS Categorical: *p* = 0.4 Continuous: *p* = 0.2	Liver			
Rejection			Health behaviours		
SIPAT Deutsch-Link et al. (27)	S *p* = 0.02	Liver	TERS Twillman et al. (18)	S *p* < 0.001	Liver
SIPAT Becker et al. (28)	NS *p* = 0.7	Liver			
Organ failure			Alcohol relapse/Substance use		
SIPAT Maldonado et al. (5)	NS *p* = 0.7	Solid organ (heart, lung, liver, kidney)	SIPAT Deutsch-Link et al. (26)	S *p* = 0.02	Liver
			TERS Twillman et al. (18)	S *p* < 0.001	Liver
Transplantion success			Compliance		
TERS Twillman et al. (18)	NS *p* = 0.2	Liver	TERS Twillman et al. (18)	S *p* < 0.001	Liver
			Immunosuppressant nonadherence		
			SIPAT Deutsch-Link et al. (27)	S *p* < 0.001	Liver

Grey background; S, statistically significant; MBHI, millon behavioral health inventory; NS, statistically nonsignificant; PACT, psychosocial assessment of transplant candidates; SIPAT, stanford integrated psychosocial assessment for transplantation; TERS, Transplant Evaluation Rating Scale.

Interestingly, all the outcomes that were nonsignificant were medical in nature (the breakdown between medical and psychosocial outcomes is shown in Table 3). It is not surprising that some of these scales were able to predict psychosocial outcomes following transplantation but were somewhat imperfect for assessing medical outcomes. These results reinforce the idea that medical outcomes are not necessarily related to psychosocial behaviour, but more importantly to other outcomes, such as hospitalizations or recurrent illnesses. This finding also highlights the importance of the multidisciplinary evaluation of the transplant candidates.

Nevertheless, it is still impressive that almost half the studies had significant association with medical outcomes. We are not aware of a reverse situation, in which a physical health scale would predict psychosocial outcomes. Anyhow, future studies are necessary to refine and maximize the predictive aspect of these tools, possibly by using specific psychosocial domains rather than total scores, as suggested by Olt et al. (31) to assess the association with post-transplant outcomes. However, it should be noted that an inherent limitation of the included studies and study populations is that post-transplant outcomes are only assessed in patients who have effectively received the transplant. Thus, investigators are testing the predictive powers of the scales in those who probably had higher scores at baseline, which may underestimate their efficacy. It is also worth mentioning that some follow-up times, such as that for SIPAT, with patients followed for one year, may be insufficient to capture certain medical outcomes, such as mortality/survival. Other teams, such as the ones evaluating TERS, have followed patients for up to 5 years, with significant results for these medical outcomes.

With these results in mind, it is clear that the involvement of a trained clinical psychologist within the transplant team remains the standard of care considering the complexity of each and every person's life conditions and preferences. The use of psychosocial questionnaires may be considered as a screening tool which helps the transplant team regarding the care of each patient. These questionnaires can be helpful in revealing major contraindications to transplant, thus guiding the need for different interventions with certain populations in order to determine if certain patients may benefit from early interventions which could result in eventual transplantation.

### 4.1. Limitations

This narrative literature review has some limitations. First, at the methodological level and inherent to our narrative design, the literature search was limited to a single database, and some relevant studies may therefore have been potentially missed. Secondly, our comparison between the different studies and scales included is limited, mainly due to differences in primary and secondary outcomes, contrastive designs (prospective vs. retrospective), marked dissimilarity in study populations (types of transplantation, demographic distributions), length of follow-up periods, types of variables and scoring (continuous vs. categorical, thresholds) and statistical analyses used to test the associations. Although we did not combine the results of the different studies as in a meta-analysis, the numerous disparities must still be considered in interpreting the results. Finally, the small number of studies per psychosocial tool assessment also calls for caution in interpreting the results, especially regarding the generalizability of the findings.

## 5. Conclusion

This narrative literature review evaluated different widely used scales assessing the psychosocial characteristics of the patients, which is an integral part of the transplantation candidacy process for solid organ transplantation, and their predictive value on postoperative outcomes. Regarding our initial interrogation as if psychosocial assessment tools for use before transplantation or mechanical circulatory support were predictive of postoperative outcomes, the definite answer should be “yes” for what they are deemed to measure, i.e., psychosocial and health behavior outcomes, and indeterminate for the physical health outcomes. Of the thirteen articles included, an association with pre-transplant scores could be found for more than half of the nineteen postoperative primary outcomes tested, with TERS and MBHI having the higher number of positive (statistically significant) studies. The overall mixed evidence towards the predictive value of the different scales remains a real challenge for transplantation teams, given the limited number of donors and the need to allocate this resource to the most suitable candidates. Consequently, evidence from well conducted clinical trials are urgently needed to empower the transplantation teams worldwide in their predictive capacity during the evaluation of the transplant candidates; our review suggests that this may only come through a multidisciplinary lens.

## References

[1] United Network for Organ Sharing. 2022 Organ Transplants Again Set Annual Records (2023). Available at: https://unos.org/news/2022-organ-transplants-again-set-annual-records/

[2] Canadian Institute for Health Information. Organ Replacement in Canada: Corr Annual Statistics (2021) [2022]. Available at: https://www.cihi.ca/en/organ-replacement-in-canada-corr-annual-statistics

[3] Canadian Institute for Health Information. Summary Statistics on Organ Transplants, Wait-Lists and Donors | Cihi (2022) [2022]. Available at: https://www.cihi.ca/en/summary-statistics-on-organ-transplants-wait-lists-and-donors

[4] Upton J. Psychosocial factors. In: Gellman MD Turner JR, editors. Encyclopedia of behavioral medicine. New York, NY: Springer New York (2013). p. 1580–1.

[5] Maldonado JR Sher Y Lolak S Swendsen H Skibola D Neri E The Stanford integrated psychosocial assessment for transplantation: a prospective study of medical and psychosocial outcomes. Psychosom Med. (2015) 77(9):1018–30. 10.1097/PSY.0000000000000241 26517474

[6] Nghiem DM Gomez J Gloston GF Torres DS Marek RJ. Psychological assessment instruments for use in liver and kidney transplant evaluations: scarcity of evidence and recommendations. J Pers Assess. (2020) 102(2):183–95. 10.1080/00223891.2019.1694527 31860362

[7] Centers for Medicare & Medicaid Services. Transplant (2021). Available at: https://www.cms.gov/medicare/provider-enrollment-and-certification/certificationandcomplianc/transplant

[8] Dew MA DiMartini AF Dobbels F Grady KL Jowsey-Gregoire SG Kaan A The 2018 ishlt/apm/ast/iccac/stsw recommendations for the psychosocial evaluation of adult cardiothoracic transplant candidates and candidates for long-term mechanical circulatory support. Psychosomatics. (2018) 59(5):415–40. 10.1016/j.psym.2018.04.003 30197247

[9] de Zwaan M Erim Y Kröncke S Vitinius F Buchholz A Nöhre M Psychosocial diagnosis and treatment before and after organ transplantation. Dtsch Arztebl Int. (2023). 120:413–9. 10.3238/arztebl.m2023.0087 37101343 PMC10437037

[10] Moher D Liberati A Tetzlaff J Altman DG Group P. Preferred reporting items for systematic reviews and meta-analyses: the prisma statement. PLoS Med. (2009) 6(7):e1000097. 10.1371/journal.pmed.1000097 19621072 PMC2707599

[11] Millon T Green CJ Meagher RB. A new psychodiagnostic tool for clients in rehabilitation settings: the mbhi. Rehabil Psychol. (1982) 27(1):23–35. 10.1037/h0090959

[12] Strack S. Essentials of millon inventories assessment. 3rd ed. Hoboken, N.J.: John Wiley & Sons (2008).

[13] Millon T Antoni MH Millon C Meagher S Grossman S. Test manual for the millon behavioral medicine diagnostic (mbmd). Minneapolis, MN: National Computer Services (2001).

[14] Olbrisch ME Levenson JL. Psychosocial assessment of organ transplant candidates. Current Status of methodological and philosophical issues. Psychosomatics. (1995) 36(3):236–43. 10.1016/S0033-3182(95)71662-0 7638310

[15] Hitschfeld MJ Schneekloth TD Kennedy CC Rummans TA Niazi SK Vasquez AR The psychosocial assessment of candidates for transplantation: a cohort study of its association with survival among lung transplant recipients. Psychosomatics. (2016) 57(5):489–97. 10.1016/j.psym.2016.05.003 27494985

[16] Maldonado JR Dubois HC David EE Sher Y Lolak S Dyal J The Stanford integrated psychosocial assessment for transplantation (sipat): a new tool for the psychosocial evaluation of Pre-transplant candidates. Psychosomatics. (2012) 53(2):123–32. 10.1016/j.psym.2011.12.012 22424160

[17] Mori DL Gallagher P Milne J. The structured interview for renal transplantation–sirt. Psychosomatics. (2000) 41(5):393–406. 10.1176/appi.psy.41.5.393 11015625

[18] Twillman RK Manetto C Wellisch DK Wolcott DL. The transplant evaluation rating scale. A revision of the psychosocial levels system for evaluating organ transplant candidates. Psychosomatics. (1993) 34(2):144–53. 10.1016/S0033-3182(93)71905-2 8456157

[19] NIH National Heart L, and Blood Institute. Study Quality Assessment Tools | Nhlbi, Nih (2019). Available at: https://www.nhlbi.nih.gov/health-topics/study-quality-assessment-tools

[20] Chacko RC Harper RG Gotto J Young J. Psychiatric interview and psychometric predictors of cardiac transplant survival. Am J Psychiatry. (1996) 153(12):1607–12. 10.1176/ajp.153.12.1607 8942458

[21] Harper RG Chacko RC Kotik-Harper D Young J Gotto J. Self-report evaluation of health behavior, stress vulnerability, and medical outcome of heart transplant recipients. Psychosom Med. (1998) 60(5):563–9. 10.1097/00006842-199809000-00009 9773759

[22] Coffman KL Brandwin M. The millon behavioral health inventory life threat reactivity scale as a predictor of mortality in patients awaiting heart transplantation. Psychosomatics. (1999) 40(1):44–9. 10.1016/S0033-3182(99)71270-3 9989120

[23] Brandwin M Trask PC Schwartz SM Clifford M. Personality predictors of mortality in cardiac transplant candidates and recipients. J Psychosom Res. (2000) 49(2):141–7. 10.1016/S0022-3999(00)00152-5 11068059

[24] Schneekloth TD Hitschfeld MJ Jowsey-Gregoire SG Petterson TM Dunlay SM Niazi SK Psychosocial risk predicts new episode depression after heart transplant. Psychosomatics. (2019) 60(1):47–55. 10.1016/j.psym.2018.06.003 30064730

[25] Schneekloth TD Hitschfeld MJ Petterson TM Narayanan P Niazi SK Jowsey-Gregoire SG Psychosocial risk impacts mortality in women after liver transplantation. Psychosomatics. (2019) 60(1):56–65. 10.1016/j.psym.2018.06.008 30122643

[26] Deutsch-Link S Weinrieb RM Jones LS Solga SF Weinberg EM Serper M. Prior relapse, ongoing alcohol consumption, and failure to engage in treatment predict alcohol relapse after liver transplantation. Dig Dis Sci. (2020) 65(7):2089–103 10.1007/s10620-019-05937-4 31707529

[27] Deutsch-Link S Weinberg EM Bittermann T McDougal M Dhariwal A Jones LS The Stanford integrated psychosocial assessment for transplant is associated with outcomes before and after liver transplantation. Liver Transpl. (2021) 27(5):652–67. 10.1002/lt.25975 33320417 PMC9531321

[28] Becker JH Shemesh E Shenoy A Posillico A Knight CS Kim SK The utility of a Pre-transplant psychosocial evaluation in predicting post-liver transplant outcomes. Prog Transplant. (2021) 31(1):4–12. 10.1177/1526924820978605 33272096 PMC7946723

[29] Baranyi A Krauseneck T Rothenhausler HB. Overall mental distress and health-related quality of life after solid-organ transplantation: results from a retrospective follow-up study. Health Qual Life Outcomes. (2013) 11:15. 10.1186/1477-7525-11-15 23391215 PMC3579763

[30] Trevethan R. Sensitivity, specificity, and predictive values: foundations, pliabilities, and pitfalls in research and practice. Front Public Health. (2017) 5:307. 10.3389/fpubh.2017.00307 29209603 PMC5701930

[31] Olt CK Thuita LW Soltesz EG Tong MZ Weiss AJ Kendall K Value of psychosocial evaluation for left ventricular assist device candidates. J Thorac Cardiovasc Surg. (2021):S0022-5223(21)00749-2. 10.1016/j.jtcvs.2021.04.065 [Epub ahead of print]. 34053742 PMC10443599

