# Supplementary material for: Psychosocial assessment tools for use before transplantation are predictive of post-operative psychosocial and health behavior outcomes: a narrative review of the literature
Source: Front Transplant. 2023 Sep 7;2:1250184. doi: 10.3389/frtra.2023.1250184 (PMC11235356; doi:10.3389/frtra.2023.1250184)
Supplement: Supplementary file 1 [file Datasheet1.docx]

**Supplementary material**

Table S1: Complete research strategy

Table S2: First MEDLINE search completed 2020-07-28

Table S3: Second MEDLINE search completed 2021-06-09

Table S4: Third and final MEDLINE search completed 2022-02-02
